# Trop2 expression contributes to tumor pathogenesis by activating the ERK MAPK pathway

**DOI:** 10.1186/1476-4598-9-253

**Published:** 2010-09-21

**Authors:** Rafael Cubas, Sheng Zhang, Min Li, Changyi Chen, Qizhi Yao

**Affiliations:** 1 Department of Molecular Virology and Microbiology, Baylor College of Medicine, One Baylor Plaza, Houston, TX 77030, USA; 2 Molecular Surgeon Research Center, Michael E. DeBakey Department of Surgery, Baylor College of Medicine, One Baylor Plaza, Houston, TX 77030, USA

## Abstract

**Background:**

Trop2 is a cell-surface glycoprotein overexpressed by a variety of epithelial carcinomas with reported low to restricted expression in normal tissues. Expression of Trop2 has been associated with increased tumor aggressiveness, metastasis and decreased patient survival, but the signaling mechanisms mediated by Trop2 are still unknown. Here, we studied the effects murine Trop2 (mTrop2) exerted on tumor cellular functions and some of the signaling mechanisms activated by this oncogene.

**Results:**

mTrop2 expression significantly increased tumor cell proliferation at low serum concentration, migration, foci formation and anchorage-independent growth. These *in vitro *characteristics translated to increased tumor growth in both subcutaneous and orthotopic pancreatic cancer murine models and also led to increased liver metastasis. mTrop2 expression also increased the levels of phosphorylated ERK1/2 mediating cell cycle progression by increasing the levels of cyclin D1 and cyclin E as well as downregulating p27. The activation of ERK was also observed in human pancreatic ductal epithelial cells and colorectal adenocarcinoma cells overexpressing human Trop2.

**Conclusions:**

These findings demonstrate some of the pathogenic effects mediated by mTrop2 expression on cancer cells and the importance of targeting this cell surface glycoprotein. This study also provides the first indication of a molecular signaling pathway activated by Trop2 which has important implications for cancer cell growth and survival.

## Background

Trop2 is a cell surface glycoprotein belonging to the *TACSTD *gene family and highly overexpressed by a variety of epithelial carcinomas with low to restricted expression in normal tissues [1-6]. Clinical data has shown a positive correlation between Trop2 expression levels and tumor aggressiveness and metastasis, and a negative correlation with overall patient survival [1-6]. Trop2 is highly conserved among species with a 79% identical amino acid composition between human and murine Trop2. This protein was initially found to be highly expressed in trophoblast cells, which arise from epithelial trophectoderm cells and become invasive, phagocytosing and displacing uterine epithelial cells. This allows for the penetration of the uterine stroma in order to establish vascular interactions with the maternal blood supply [7,8]. Trop2 expression has also been observed in murine and human prostate basal cells with stem cell characteristics [9]. Basal stem progenitor cells with high Trop2 expression were shown to give rise to basal, luminal and even neuroendocrine cells *in vivo*. A similar behavior has also been reported in hepatic oval cells which are considered facultative hepatic stem cells and shown to express Trop2 [10]. It thus appears that Trop2 provides crucial signals for cells with a requirement for proliferation, survival and invasion such as trophoblast cells or cells with progenitor-like characteristics. These same characteristics might be conferred to cancer cells by overexpression of this surface glycoprotein.

Trop2 has recently been identified as an oncogene leading to the invasiveness and tumorigenesis of colon cancer cells, but the underlying signaling mechanisms activated by this protein are still unknown [11]. It has been shown that cross-linking this protein with antibodies results in a significant rise in intracellular calcium [Ca^2+^] from internal stores which could have a significant effect on the activation and progression of the cell cycle as well as activation of other signaling pathways [12-15]. The cytoplasmic tail of Trop2 appears to play an important role in signaling. One study has shown the presence of a phosphatidylinositol 4,5-bis phosphate (PIP_2_)-binding sequence highly homologous to that of gelsolin [16]. Within this sequence there is a conserved serine residue which is phosphorylated by protein kinase C (PKC) [17]. Thus, PKC and mitogen-activated protein kinases (MAPKs) including ERK1/2 may be involved in Trop2 induced tumor cell growth [17,18].

The purpose of this study was to determine the effects of murine Trop2 expression (mTrop2) in cancer cells and to start delineating the pathways activated by this molecule. We found that mTrop2 expression resulted in increased cell proliferation at low serum concentrations with an increased percentage of cells entering S phase. Expression of mTrop2 also led to increased cell migration, foci formation and anchorage independent growth and translated to increased tumor growth in both subcutaneous and orthotopic tumor models. mTrop2 expression also led to increased liver metastasis as well as increased levels of phosphorylated p42/p44^MAPK ^(ERK1/ERK2) which is a master regulator of the G_1_- to S-phase transition [18,19]. This translated to a rise in cyclin D1 and cyclin E protein levels with a downregulation of p27. This study provides new evidence that Trop2 contributes to tumor pathogenesis at least in part by activating the ERK1/2 MAPK pathway which has important implications for a variety of cellular pathways as it can affect cancer cell proliferation, migration, invasion and survival [18,20-22].

## Results

### Expression of mTrop2 increases cell proliferation at low serum concentrations

**Figure 1:**
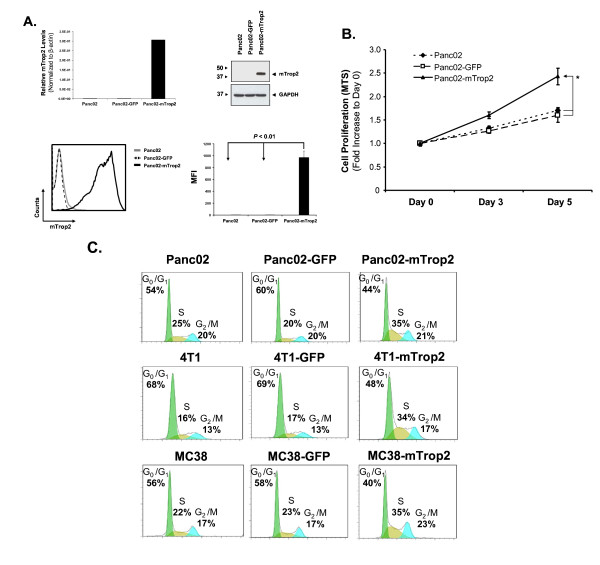
**Expression of mTrop2 can promote cell proliferation and cell cycle progression**. (a) Stable murine pancreatic adenocarcinoma cells (Panc02) expressing either mTrop2 (Panc02-mTrop2) or GFP (Panc02-GFP) were generated. The increase in mTrop2 mRNA levels in Panc02-mTrop2 cells was measured by quantitative real-time RT PCR. The *y *axis represents the normalized mTrop2 expression relative to β-actin expression. The protein levels of mTrop2 were determined by Western blot analysis and its membrane incorporation by flow cytometry. (b) Panc02-mTrop2 and control cells were seeded in 96-well plates (1 × 10^3 ^cells/well) and serum-starved for 24 h before changing to growth medium containing 0.2% FBS. MTS assay was performed after breaking serum starvation. Relative increase in viability was measured by dividing the viability at one time point by the viability of the same cell at day 0. Absorbance means ± SD (n = 5) are shown. *P < 0.01. (c) Cell cycle analysis. After 24 h of serum starvation, cells were released by the addition of 2% FBS for 4 h (Panc02 and 4T1 cells) or 8 h (MC38 cells). Cells were collected, fixed, stained with propidium iodide and analyzed for cell cycle phase distribution. Data shown is representative from three independent experimental repeats.

In order to elucidate whether mTrop2 expression has any effect on the growth of cancer cells we generated stable murine pancreatic adenocarcinoma cells (Panc02) expressing mTrop2 (Panc02-mTrop2) since this cell line does not naturally express this surface glycoprotein. A control cell line expressing GFP (Panc02-GFP) was also generated. To determine the function of mTrop2, Panc02-GFP and the parental cell line Panc02 were used as controls in all assays. As shown in Fig. 1A, stable Panc02-mTrop2 cells express mTrop2 as determined by real-time quantitative PCR and immunoblotting and this expression is present on the cell surface as demonstrated by flow cytometry using an anti-mTrop2 monoclonal antibody. All three cell lines were then used in a proliferation assay to assess any difference in the growth rate capabilities of these cells. The results showed that Panc02-mTrop2 cells had a significant increase in proliferation at low serum concentrations when compared to normal Panc02 or Panc02-GFP cells (*P *< 0.01; Fig. 1B). Panc02-mTrop2 cells proliferated 2.7 times faster than Panc02-GFP cells at day 5. It is important to note that expression of mTrop2 did not appear to affect proliferation at high serum concentrations and this was only evident when low serum levels were used (data not shown). To obtain a more comprehensive understanding of the effect mTrop2 had on cell proliferation, we examined the cell cycle progression of Panc02, Panc02-GFP and Panc02-mTrop2 cells by propidium iodide staining and flow cytometry analysis. In order to confirm that the effect on cell cycle progression conferred by mTrop2 is not restricted to Panc02 cells, but rather a generalized effect, we included stable GFP (4T1-GFP) and mTrop2 expressing (4T1-mTrop2) murine breast cancer (4T1) and murine colorectal adenocarcinoma (MC38) cells (MC38-GFP and MC38-mTrop2). As depicted in Fig. 1C, there was an increase in the percentage of cells entering S phase after releasing serum starved cells with 2% serum-containing medium in all cell lines expressing mTrop2. This increase in cells entering S phase came with a reduction in the population of cells in the G_0_/G_1 _phase. The percentage of cells entering S phase in the mTrop2 group was 35% which was about 10% (35% vs. 25%) and 15% (35% vs. 20%) higher when compared to the Panc02 and Panc02-GFP groups, respectively. A similar trend was also observed in 4T1-mTrop2 and MC38-mTrop2 cells where there was a significant increase in the percentage of cells entering S phase when compared to the control cells. These results demonstrate that mTrop2 expression leads to increased cell growth by inducing a faster progression into the synthesis phase of the cell cycle.

### Expression of mTrop2 enhances cell migration, foci formation and anchorage- independent growth

**Figure 2:**
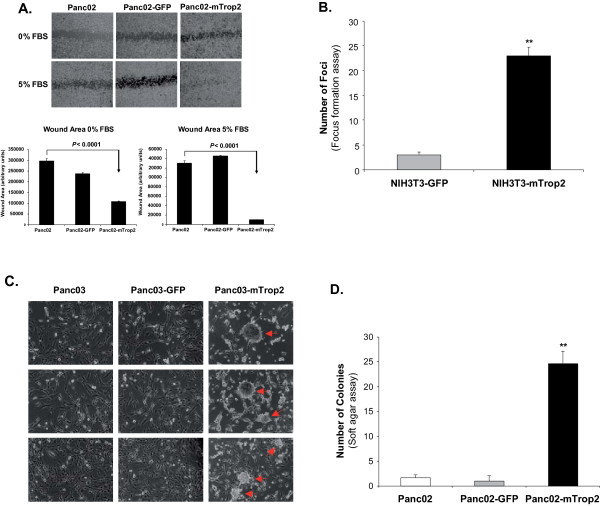
**Expression of mTrop2 leads to increased migration, foci formation and anchorage-independent growth**. (a) Panc02-mTrop2 and control cells were serum starved for 24 h followed by the addition of media containing either 0% or 5% FBS. Wounds were generated in confluent cell monolayers utilizing a sterile pipette tip. Pictures were taken 24 h after the wound insult. A representative field for each cell line is shown. (b) The expression of mTrop2 led to a loss of contact inhibition and foci formation. Foci greater than 1 mm were counted after 16 days. Mean ± SD is shown (*n = 3*). ** P < 0.0001. (c) Expression of mTrop2 on the more indolent murine pancreatic adenocarcinoma cell line, Panc03, also led to increased foci formation (arrows). (d) Panc02-mTrop2 cells acquire an enhanced ability to grow in soft agar. Panc02-mTrop2 and control cells were plated in 6-well plates as described in the Methods section. Colonies were measured after 72 h to determine the increased rate of colony formation in Panc02-mTrop2 cells. Results are shown as mean ± SD (*n = 3*). *P < 0.001.

Increased migration is a characteristic of aggressive cancer cells. To determine whether mTrop2 expression could result in increased cell migration, we performed a monolayer wound healing assay. Panc02 cells are naturally aggressive and tend to migrate at accelerated rates. However, expression of mTrop2 resulted in a further increase in the rate of migration when compared to the parental and GFP control cell lines at both 0% and 5% serum concentrations (*P *< 0.0001) (Fig. 2A). In 5% serum conditions the induced wound was barely visible in the Panc02-mTrop2 group after 24 hours. This increase in migration was also observed in the absence of serum suggesting that mTrop2 might have an intrinsic ability to foster cell migration without the presence of growth factors.

The generation of foci represents a loss of contact inhibition or the ability to maintain cell growth and movement despite contact with surrounding cells. To determine whether ectopic expression of mTrop2 could transform cells and confer loss of contact inhibition, we transfected NIH3T3 cells with GFP- (NIH3T3-GFP) or mTrop2- (NIH3T3-mTrop2) containing plasmids. These cells were then allowed to grow in 6-well plates until foci greater than 1 mm were apparent. As shown in Fig. 2B, mTrop2 expression led to an 11.5-fold increase in the number of foci generated when compared to the GFP control group (*P *< 0.0001). This shows that transfection with a plasmid expressing mTrop2 is sufficient to induce the transformation of NIH3T3 cells. This ability of mTrop2 to induce foci formation was also observed when mTrop2 was expressed in the more indolent murine pancreatic adenocarcinoma cell line Panc03 (Fig. 2C).

To further study the phenotypic changes conferred by mTrop2 on cancer cells, we evaluated the ability of this protein to increase the rate of soft agar colony formation on Panc02 cells. As shown in Fig. 2D, mTrop2 expression resulted in a 12.5-fold increase in the number of colonies formed at a very early time point. This represents a significant change in the growth rate capability of these cells in soft agar and an ability to proliferate under such stringent conditions. mTrop2 is thus capable of increasing the proliferative capacity and aggressiveness of tumor cells and might also be providing certain survival signals.

### Expression of mTrop2 correlates with increased tumor growth

**Figure 3:**
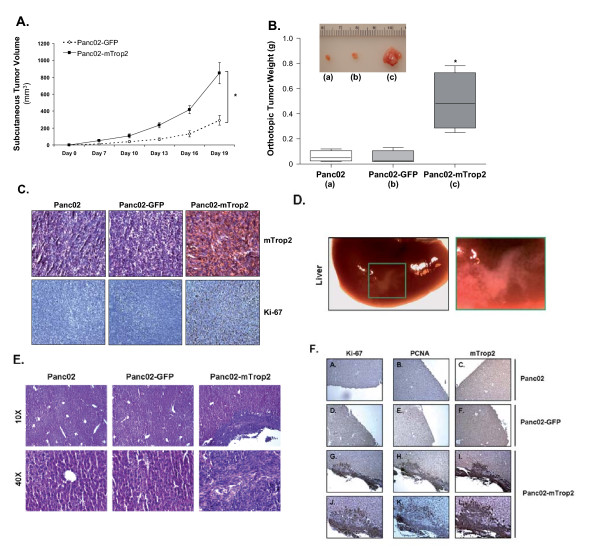
**mTrop2 expression increases tumorigenesis in subcutaneous and orthotopic tumor models**. (a) Nu/nu mice were inoculated subcutaneously with 2 × 10^5 ^Panc02-GFP or Panc02-mTrop2 cells. Tumor size was monitored for 22 days with digital calipers and volumes reported as mean ± SE (*n *= 5). *P < 0.01. (b) For the orthotopic model, nude mice were inoculated with 5 × 10^4 ^Panc02, Panc02-GFP or Panc02-mTrop2 cells in the pancreas. Mice were monitored daily and euthanized after 14 days; data are reported as mean ± SD (*n *= 6). *P < 0.01. (c) Expression of mTrop2 led to an increase in tumor size and cell proliferation. Immunohistochemistry of paraffin embedded tumor tissues show expression of mTrop2 and Ki-67. (d) Panc02-mTrop2 cell inoculation results in liver metastasis as depicted in the square and blow out picture. (e) H&E staining shows normal liver cell structure in Panc02 and Panc02-GFP inoculated mice and liver metastasis with aberrant liver cell structure in Panc02-mTrop2 inoculated mice (10× low magnification and 40× high magnification) (f) Immunohistochemistry for Ki-67, PCNA and mTrop2 in livers from Panc02 (A-C), Panc02-GFP (D-F) and Panc02-mTrop2 (G-I at 10× and J-L at 20× magnification) inoculated mice. All images shown are representative of group.

We have shown that mTrop2 expression in tumor cells can lead to an increase in cell proliferation, migration and aggressiveness in various *in vitro *studies. In order to investigate the effects of mTrop2 expression in an *in vivo *setting, we inoculated Panc02-GFP and Panc02-mTrop2 cells subcutaneously into the left flank of immunodeficient nude mice to compare their overall growth rate. As observed in Fig. 3A, Panc02-mTrop2 cells showed a significant increase (3-fold) in tumor growth over GFP control cells (*P *< 0.01). Since a subcutaneous setting differs from an orthotopic environment, we wanted to confirm whether the observed increase in tumor growth rate was also reproducible in more realistic growth conditions and whether there was any effect on the metastatic potential of these murine pancreatic cancer cells. To achieve this, Panc02, Panc02-GFP or Panc02-mTrop2 cells were inoculated into the tail of the pancreas in immunodeficient mice. Tumors were allowed to grow for 2 weeks at which point mice were euthanized and the tumors extracted for further characterization. As shown in Fig. 3B, mice inoculated with Panc02-mTrop2 cells showed an 8.3- and 10-fold increase in tumor weight with respect to mice inoculated with control Panc02 or Panc02-GFP cells, respectively (*P *< 0.01). The extensive difference in tumor size can be visualized in Fig. 3B. Immunohistochemistry was used to confirm the expression of mTrop2 in pancreatic tumor tissues from mice inoculated with Panc02-mTrop2 cells. The expression of mTrop2 correlated with increased expression of the proliferation marker Ki-67. One third of the mice from the Panc02-mTrop2 group also showed indications of liver metastasis (Fig. 3D-E). Further staining with Ki-67, PCNA and mTrop2 confirmed the presence of mTrop2 expressing tumor cells inside the liver which also showed increased Ki-67 and PCNA expression (Fig. 3F). These results corroborate our *in vitro *data which shows that mTrop2 expression can increase the growth capacity and aggressiveness of tumor cells.

### mTrop2 expression increases activation of the ERK1/2 MAPK pathway

**Figure 4:**
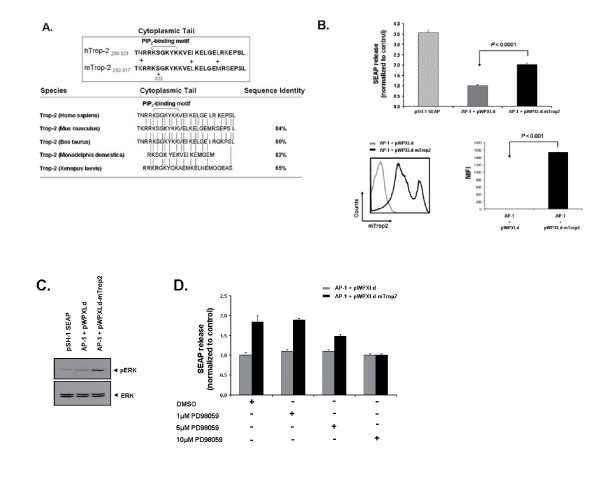
**Trop2 is highly conserved among species and mTrop2 expression can stimulate the AP-1 transcription factor**. (a) The percentage of sequence identity between the human and murine cytoplasmic tail of Trop2 is 84%. A similar conservation level is observed among other species. "+" indicates a.a. difference. (b) Expression of mTrop2 leads to activation of the AP-1 transcription factor. 293T cells were transiently co-transfected with an AP-1 SEAP reporter construct together with a vector control (pWPXLd) or mTrop2 expressing vector (pWPXLd-mTrop2). As a positive control a pSH-1 SEAP construct was used. Media was collected and processed for SEAP assays and a representative result from three separate experiments done in triplicate is shown as mean ± SD. The negative control was assigned a value of 1, and the other data was normalized to this value. At the time of media collection co-transfected 293T cells show a high level of mTrop2 expression as shown by flow cytometry. (c) Cell lysate from transfected 293T cells used in the AP-1 SEAP assay was harvested and used for immunoblotting to detect the levels of total and phosphorylated ERK1/2. (d) 293T cells were transiently co-transfected with an AP-1 SEAP reporter construct together with pWPXLd or pWPXLd-mTrop2. Cells were subsequently treated with media containing different concentrations of the MEK1 inhibitor PD98059 or DMSO. Media was collected and processed for SEAP assays. Results are shown as mean ± SD.

Little is known about the signaling pathways activated by Trop2. Earlier work has shown that this protein increases the level of intracellular calcium which could potentially have an effect on a variety of proteins involved in cell signaling mechanisms [12]. Other work has demonstrated that the cytoplasmic tail which contains a conserved PIP_2_-binding motif and a serine residue phosphorylated by protein kinase C (PKC) might be essential for signaling [16,17]. The cytoplasmic tail for both murine and human Trop2 is highly conserved with an 84% sequence identity and only a three amino acid difference (Fig. 4A). A similar degree of conservation is also observed for different species alluding to the likely importance the cytoplasmic tail has for signaling and suggesting a maintenance of Trop2 functions throughout different species. Despite these initial observations, the mechanism of action for this protein is still unknown.

The mitogen-activated protein kinase (MAPK) pathways can be activated by a variety of stimuli leading to the activation of multiple programs like cell proliferation and motility, differentiation, as well as survival and apoptosis [21]. Due to the apparent involvement of mTrop2 in cell growth and aggressiveness we wanted to determine whether there was induction of MAPK signaling. To test for the induction of MAPK pathways we used an activator protein 1 (AP-1) secreted alkaline phosphatase (SEAP) reporter assay as this transcription factor lies downstream of MAPK activation. As shown in Fig. 4B, 293T cells transfected with an AP-1 SEAP reporter construct together with a lentiviral vector containing the mTrop2 gene (pWPXLd-mTrop2) led to a significant increase in SEAP release when compared to the vector control group (pWPXLd) signifying the induction of AP-1 transcription. After transfection and at the time of the assay 293T cells transfected with the mTrop2 expression construct showed a high level of mTrop2 expression as demonstrated by flow cytometry (Fig. 4B). These results indicate that expression of mTrop2 can lead to the activation of MAPK signaling which results in the induction of the AP-1 transcription factor.

In our cell cycle analysis, we observed an increase in the percentage of cells entering S phase. This transition from G_1_- to S-phase is largely mediated by the sustained activation of ERK1/2 during the late stages of the G_1 _phase [23]. This MAPK pathway can be further stimulated by an increase in Ca^2+ ^and activated ERK can increase AP-1 activity via induction of *c-fos *[24,25]. It is therefore possible that the ERK MAPK pathway is implicated in mTrop2 signaling. To determine whether induction of the AP-1 transcription factor was mediated preferentially by ERK and not JNK or p38 signaling, cell lysates from 293T cells used in the AP-1 SEAP assays were harvested and used for immunoblotting to detect the levels of total and phosphorylated ERK1/2. As shown in Fig. 4C, 293T cells transfected with the mTrop2 expression construct showed a higher level of phosphorylated ERK when compared to the vector and pSH-1 SEAP control cell lysates. To corroborate that the change in SEAP activity mediated by AP-1 and observed in 293T cells expressing mTrop2 was due to ERK signaling, cells were treated with different concentrations of the MEK1 inhibitor PD98059 which lies upstream of ERK. As observed in Fig. 4D, increasing concentrations of PD98059 led to a reduction in AP-1 mediated SEAP release confirming the involvement of ERK singling in the induction of AP-1 transcription following mTrop2 expression. The observed changes on SEAP release were not due to cell cytotoxicity as cell viability was not affected by the different concentrations of PD98059 used (data not shown).

**Figure 5:**
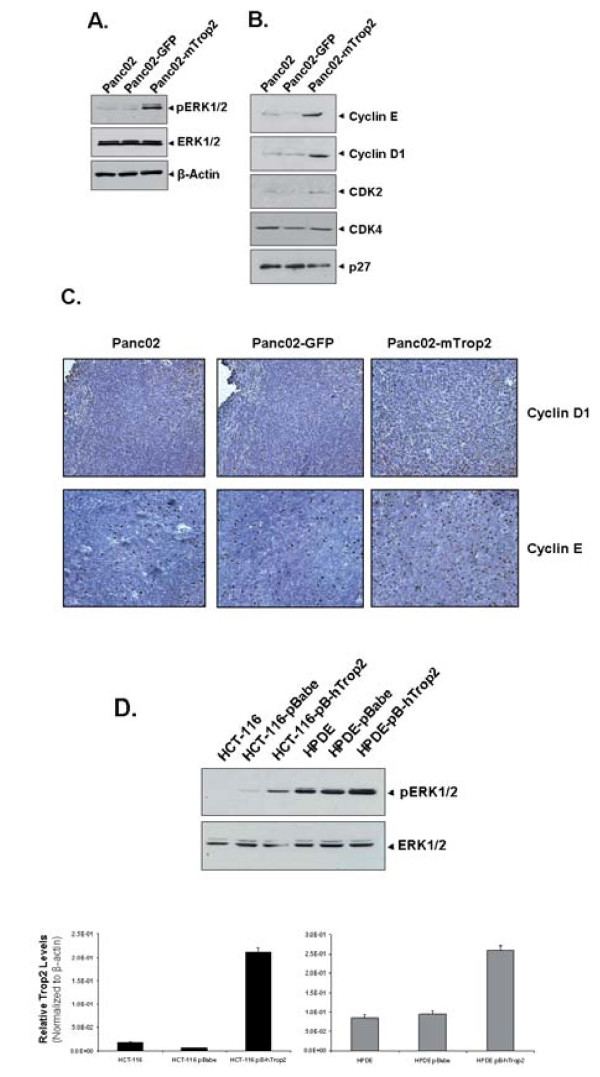
**mTrop2 expression stimulates the ERK MAPK pathway**. (a) Subconfluent cells were used to prepare cell lysates and 60 μg of protein were used for immunoblotting using antibodies against phosphorylated and total ERK. (b) ERK downstream targets like cyclin E, cyclin D1 and CDK2 show increased expression in mTrop2 expressing cells while p27 is downregulated. (c) Increased expression of cyclin D1 and cyclin E was also observed in tumor tissues from mice inoculated with Panc02-mTrop2 cells, when compared to control Panc02 or Panc02-GFP tumor tissues. Images shown are representative of group. (d) Cell lysates from stable human colorectal cancer (HCT-116) and human pancreatic ductal epithelial (HPDE) cells overexpressing human Trop2 were also used for the detection of activated ERK. Increase in hTrop2 mRNA level in HCT-116 and HPDE cells was measured by quantitative real-time RT PCR. The *y *axis represents the normalized hTrop2 expression relative to β-actin expression.

To continue investigating the activation of ERK signaling by mTrop2 expression, we examined the levels of phosphorylated ERK1/2 in Panc02, Panc02-GFP and Panc02-mTrop2 cells and found that the phosphorylated levels of ERK1/2 were significantly higher in Panc02-mTrop2 cells (Fig. 5A). The levels of cyclin D1 and cyclin E were also significantly increased in both Panc02-mTrop2 cells and Panc02-mTrop2 tumors as shown by western blot analysis and immunohistochemistry (Fig. 5B, C). These two molecules are downstream targets of the ERK MAPK pathway and are involved in the termination of the G_0_-G_1 _cell cycle arrest and initiation and progression of the S phase. CDK2, which interacts with cyclin E during the initiation and progression of the S phase, was also increased in Panc02-mTrop2 cells. This increase was not observed for CDK4 one of the CDKs which interacts with cyclin D (Fig. 5B). The level of the CDK inhibitor p27, which acts as an inhibitor of cell proliferation, was also decreased in Panc02-mTrop2 cells (Fig. 5B).

We also wanted to confirm whether this activation of ERK could be observed in a human pancreatic ductal epithelial cell line (HPDE) overexpressing human Trop2 (hTrop2) since pancreatic adenocarcinoma, which represents 95% of pancreatic cancers, is thought to arise from mutations in pancreatic ductal epithelial cells [26]. A human colorectal cancer cell line (HCT-116) overexpressing hTrop2 was also included. As shown in Fig. 5D, overexpression of hTrop2 in these cell lines led to an increase in the phosphorylated levels of ERK1/2. These results indicate that the ERK signaling pathway is indeed activated by Trop2. Whether the activation of the ERK pathway is mediated indirectly by an increase in intracellular calcium or directly through protein interactions via the cytoplasmic tail still needs to be elucidated.

## Discussion

In the current study, we used murine Trop2 to investigate the effects of its expression on murine pancreatic cancer cell proliferation and tumor growth. We showed that mTrop2 expression in the murine pancreatic cancer line (Panc02) led to an increased number of cells entering S phase which resulted in increased cell growth at low serum concentrations. Similarly, there was an enhanced ability of cells expressing mTrop2 to migrate even without the presence of serum in the media. This low requirement for serum might be indicative that Trop2 transduces a survival signal in a growth-factor independent manner. Trop2 expression also led to foci formation in NIH3T3 cells showing that expression of this protein can lead to a loss of contact inhibition. Further evidence for the role of mTrop2 in cell proliferation and/or survival was observed in the improved ability of Panc02 cells to form colonies in soft agar. Panc02 cells normally form colonies in soft agar, but expression of mTrop2 increased the rate of colony formation and by day 3 there were already on average 25 colonies compared to 1 colony for the vector control group and these colonies did not arise from cell clumping. Such *in vitro *characteristics were further maintained in subcutaneous and orthotopic tumor models where Panc02-mTrop2 cells led to a significant increase in tumor growth and metastatic rate. It is therefore evident that mTrop2 increases the growth, aggressiveness and possibly survival signals inside the cell.

By using an AP-1 SEAP reporter assay as well as cell lysates from control and mTrop2 expressing cells, we were able to delineate an initial signaling pathway activated by mTrop2. mTrop2 expressing cells showed an increase in the levels of phosphorylated ERK1/2 suggesting an activation of this MAPK pathway. Cell division is a complex process involving an intricate network of regulatory pathways [18]. One of these regulatory pathways is the ERK1/2 mitogen-activated protein kinase pathway which transduces extracellular signals into intracellular responses and is necessary for G_1_- to S-phase transition. This MAPK pathway can be activated by a variety of stimuli including mitogens, cytokines, and growth factors which induce a transient rise in intracellular calcium [Ca^2+^] from both internal and external stores. The cross-linking of Trop2 has previously been shown by others to result in a significant rise in cytoplasmic calcium [Ca^2+^] and this could in turn be activating the MAPK pathway through activation of PKC and/or Ca^2+^/calmodulin-dependent protein kinase II (CaMKII), both of which can modulate the ERK pathway [12,27,28]. These two proteins are activated by an increase in Ca^2+ ^and CaMKII can bind and phosphorylate MEK1 leading to the activation of ERK [27,29]. The link between Trop2-induced calcium increase and activation of the ERK1/2 MAPK pathway has yet to be established.

It is important to note that downstream activation of AP-1 can be mediated not only by ERK activation, but also by JNK or p38 MAPKs [30]. In this study we only focused on ERK activation due to the observed changes on cell growth and cell cycle progression observed following mTrop2 expression as well as the preferential involvement of ERK in the AP-1 SEAP assays. However, it is possible that crosstalk with the other MAPK pathways is taking place upstream of AP-1 as this transcription factor serves as a connecting node, linking various signal transduction pathways [31]. Trop2 could therefore be affecting other MAPK pathways to some degree. Nonetheless, ERK signaling can activate AP-1 which can play an important role in cell proliferation, apoptosis, differentiation, cancer cell invasion and has been shown to regulate cyclin D1 and E2F in breast cancer cells [31].

Upon phosphorylation of the activation loop residues of p44/p42 by MEK2, there is subsequent activation of downstream targets which include transcription factors and genes important for the cell cycle such as *cyclin D *and *cyclin E *[32]. In the current study, an increase in cyclin D1 and cyclin E expression was indeed observed in Panc02 cells expressing mTrop2. Cyclin D1 partners with CDK4 and CDK6 in the early to mid-G_1 _phase to phosphorylate and inactivate the retinoblastoma protein (pRB). The inactivation of pRB is also mediated by the cooperation of cyclin E/CDK2 both of which showed increased expression in mTrop2 expressing cells. Cyclin D1 and cyclin E are both key regulators of the G_1_- to S-phase transition and have been implicated with tumorigenesis and metastasis [33,34]. The cyclin-dependent kinase inhibitor 1B, also known as p27, which binds to and prevents the activation of cyclin D1-CDK4 or cyclin E-CDK2 complexes, was also downregulated in mTrop2 expressing cells corroborating a progression of the cell cycle [35].

Apart from a role in cell-cycle progression cyclin D1 could also be providing additional signals independent of CDK4/6 which are also implicated in tumorigenesis such as interaction with both FOXO1 and FOXO3a to inhibit anoikis [33]. This inhibition could allow cells not only to survive and proliferate, but also to metastasize in the absence of an extracellular matrix support, something that was observed in our anchorage-independent growth assay and orthotopic murine model where Panc02-mTrop2 cells showed an improved capacity for anchorage-independent growth and an increased metastatic potential [36]. Heightened ERK activity could also induce the phosphorylation of FOXO3a at residues S294, S344 and S425 promoting its cytoplasmic localization and proteasomal degradation following ubiquitination by MDM2 [37]. This interaction between the ERK pathway and FOXO3a has been shown to promote cell growth and tumorigenesis, but whether Trop2 induced activation of ERK results in FOXO3a degradation still needs to be determined [38]. Activation of ERK1/2 could also be providing anti-apoptotic signals thus promoting the survival of tumor cells [20,39].

The majority of the experiments presented here focused on the use of the murine pancreatic cancer cell line Panc02 and expression of the murine homolog of Trop2. Even though Trop2 is highly conserved among species and similarities between murine and human Trop2 suggest a conservation of protein structure and a conservation of intracellular signaling, there is a possibility that murine and human Trop2 might induce different effects in murine and human cancer cells respectively. It is therefore important to confirm the results presented here in multiple human pancreatic cancer cell lines expressing human Trop2.

It is evident that Trop2 expression increases the level of phosphorylated ERK1/2 which has downstream effects on various cellular functions. Inhibition of this pathway could have a significant effect on tumor cell growth. Targeting this MAPK pathway with the use of chemical inhibitors could potentially be used as a way to counteract at least some of the oncogenic effects mediated by this cell-surface glycoprotein and potentially affect Trop2 expressing tumor cells at metastatic sites. Inhibitors of the ERK pathway have already entered clinical trials as potential therapeutic agents, but ERK inhibitors can block a number of signals upstream of ERK [40,41]. In the case of pancreatic cancer, more than 90% of pancreatic adenocarcinomas show mutations in the *KRAS *gene which result in constitutively active Ras, which can affect the activation of the ERK MAPK pathway [42-44]. Therefore targeting ERK in pancreatic cancer patients will not specifically block signals from Trop2, but would rather block a number of signals which result in the activation of ERK such as those induced by *KRAS *mutations. The use of ERK inhibitors in pancreatic cancer patients could therefore have no specific association with Trop2 and a specific inhibitor targeting Trop2 mediated signals would be highly desirable and could potentially augment the effects of ERK MAPK pathway inhibitors like PD0325901 and AZD6244 on pancreatic cancer cells. Further investigation into the signaling mechanisms and protein interactions mediated by Trop2 could lead to a better understanding of the important role this protein plays in cancerous cells. Precise protein interactions with its cytoplasmic tail as well as interactions with its extracellular region and studies aimed at determining the ligand for Trop2 could aid in the development of compounds specifically targeting Trop2 functions. The association of this molecule with prostate and hepatic oval cells displaying stem-cell characteristics hints to the possibility that Trop2 could potentially be present and used as a marker for cancer stem cells as has recently been reported for human prostate cancer [45]. Whether Trop2 plays a role in deregulating characteristic stem cell proliferation and differentiation pathways such as Notch, hedgehog and Wnt deserves further attention. If Trop2 is indeed expressed by cancer stem cells, targeting and thoroughly understanding the mechanistic pathways affected by this molecule becomes of further importance.

## Conclusions

In this study we show that mTrop2 expression results in increased tumor cell growth, apparent aggressiveness and metastatic potential. Expression of this cell surface glycoprotein also led to activation of the ERK MAPK pathway promoting cell cycle progression by increasing the levels of cyclin D1 and cyclin E in the murine pancreatic adenocarcinoma cell line Panc02. Activation of the ERK MAPK pathway has important implications not only for tumor growth, but via cross-talk with other signaling pathways and molecules could be involved in invasion, metastasis and survival. The overall behavior of Trop2 could also be affected by the specific cancer cell line used such that future experiments should focus on a panel of cell lines from different types of cancer. Hopefully this study will incite additional research on this highly important molecule so that we can soon have a more thorough understanding of the pathways affected by this cell-surface glycoprotein which could translate into the development of novel therapeutics that could be used against a variety of epithelial cancers overexpressing Trop2.

## Methods

### Cell culture and antibodies

Panc02 murine pancreatic adenocarcinoma cells were originally established by Corbett et al. by implanting cotton threads into the pancreas of C57BL/6 mice which were impregnated with 3-methylcholanthrene [46]. These cells were a kind gift from Dr. Sabry el-Naggar (Medical University of South Carolina) and were maintained in DMEM supplemented with 5% fetal bovine serum (FBS) (HyClone), 100 U/ml penicillin and 100 μg/ml streptomycin (Gibco). NIH3T3 and 4T1 cells were a kind gift from Dr. Paul Ling and Dr. Adrian Lee (Baylor College of Medicine) and were maintained in DMEM supplemented with 10% FBS, 100 U/ml penicillin and 100 μg/ml streptomycin (complete DMEM). MC38 murine colorectal adenocarcinoma cells were a kind gift from Dr. John C. Morris (National Cancer Institute). These cells were maintained in RPMI 1640 medium supplemented with 10% FBS, 100 U/ml penicillin and 100 μg/ml streptomycin. Cells were grown at 37°C in 5% CO_2_. The human colonic epithelial cell line HCT-116 was obtained from ATCC (CCL-247) and maintained in complete DMEM media. Human pancreatic ductal epithelial cells (HPDE) previously described by Furukawa et al. were maintained in keratinocyte serum-free (KSF) medium supplemented with bovine pituitary extract and epidermal growth factor (Gibco) [47].

The following antibodies and dilutions were used: anti-p44/42 MAPK (Thr202/Tyr204) 1:1000 (Cell Signaling, 9101), anti-cyclin D1 1:500 (Santa Cruz, sc-20044), anti-p27 1:1000 (Cell Signaling, 2552), anti-CDK2 1:1000 (Cell Signaling, 2546), anti-CDK4 1:1000 (Cell Signaling, 2906), anti-cyclin E 1:500 (Upstate Cell Signaling Solutions, 07-687), goat anti-rabbit IgG, HRP-linked 1:2000 (Cell Signaling, 7074) and goat anti-mouse IgG, HRP-linked 1:2000 (Cell Signaling, 7076).

### Stable cell lines

To generate stable Panc02 cells expressing mTrop2, full-length mTrop2 cDNA (BC117720) was cloned into the lentiviral vector pWPXLd (Addgene). Lentivirus harboring the mTrop2 gene was generated by cotransfecting the 2^nd ^generation packaging vector psPAX2 (Addgene), the envelope containing plasmid pMD2.G (Addgene) and pWPXLd-mTrop2 into 293FT cells. For control lentivirus normal pWPXLd was utilized. Viral supernatants were collected, filtered, concentrated and used to infect Panc02 cells. Cells were selected based on their expression of mTrop2 or eGFP as measured by real-time RT-PCR, immunoblotting and flow cytometry. This procedure was used for the other murine cell lines as well (4T1 and MC38).

For the generation of stable HCT-116 and HPDE cells overexpressing human Trop2 (hTrop2) a pBabe-hTrop2 vector was utilized. This vector was a kind gift from Dr. Loren Michel (Washington University School of Medicine). Retrovirus harboring either the pBabe or pBabe-hTrop2 constructs were generated and used for the infection of cells followed by selection with puromycin.

### Immunohistochemistry

For immunohistochemical staining tumor and liver tissue samples were extracted and fixed overnight in formalin. The next day samples were washed in 70% ethanol and embedded in paraffin. Sections (5 μm) were then cut and mounted onto glass slides followed by overnight incubation at 55°C. The tissues were then deparaffinized and rehydrated with xylene and graded alcohol series. Antigen retrieval was performed by using 10 mM sodium citrate buffer (pH 6) for 20 min. Endogenous peroxidases were quenched by incubating slides for 20 min in methanol containing 30% hydrogen peroxide. Samples were then blocked for 1 hr followed by overnight incubation of primary antibodies at 4°C. The antibody dilutions used were: anti-murine Trop2 1:40 (R&D Systems, AF1122), anti-Ki-67 1:1000 (Novacastra, United Kingdom), anti-PCNA 1:500 (Santa Cruz, sc-7907), anti-cyclin D1 1:500 (Santa Cruz, sc-20044) and anti-cyclin E 1:500 (Upstate Cell Signaling Solutions, 07-687). Slides were then washed in PBS followed by incubation with biotinylated secondary antibodies for 30 min. Stain was visualized by incubating slides for 30 min with ABC reagent followed by diaminobenzidine (DAB) treatment for 2-5 min (Vector Laboratories).

### SEAP reporter assay

Partially confluent 293T cells (70-80%) were co-transfected with 200 ng of AP-1 secreted alkaline phosphatase (SEAP) reporter gene plasmid DNA (pAP-1 SEAP), 500 ng of expression vector DNA (pWPXLd, pWPXLd-mTrop2) or positive control vector (pSH-1 SEAP) with Fugene HD transfection reagent (Roche) in 24-well plates. After 24 hours media was removed and serum-free media added to each well. The next day media was collected and assayed for SEAP activity using a FLUOstar Optima fluorescence plate reader (BMG Labtech).

### Proliferation assays

For the proliferation assay, 2000 cells/well were seeded in flat-bottom 96-well plates in complete DMEM containing 5% FBS. The next day, cells were serum-starved for 24 h followed by the addition of 0.2% FBS. Cells were cultured for 3 or 5 days, at which point 20 μl of 3-(4,5-dimethyl-thiazol-2yl)-5-(3-carboxymethoxyphenyl)-2-(4-sulfophenyl)-2*H*-tetrazolium (MTS) (Promega, Madison, WI) was added to each well and incubated at 37°C for 1.5 h. Absorbance was recorded at 490 nm with an EL-800 universal microplate reader (Bio-Tek Instruments, Winooski, VT).

For the proliferation assay in the presence of the MEK1 inhibitor PD98059 (Calbiochem, San Diego, CA), serum starvation was released by the addition of DMEM containing 0.2% FBS and PD98059 (1 μM) for 4 h. After incubation cells were carefully washed twice and kept in DMEM with 0.2% FBS. Cells were cultured for 3 days followed by MTS analysis.

### Cell cycle analysis

Cells were serum-starved for 24 h followed by the addition of media containing 2% serum and collected after 4 or 8 h. Cells were harvested and processed using the CycleTEST PLUS DNA reagent kit (Becton Dickinson, Franklin Lakes, NJ) following the manufacturer's instructions. Briefly, cells were washed three times with buffer containing sodium citrate, DMSO and sucrose. Cells were subsequently incubated for 10 min each in solution A (enzymatic digestion of cell membranes and cytoskeletons), solution B (inhibit trypsin activity and digest RNA) and solution C (stoichiometric binding of propidium iodide to DNA at a final concentration of 125 ug/ml). Cells were analyzed by flow cytometry using a FACSCalibur (Becton Dickinson) and FlowJo ver. 7.2.1 (Tree Star, Ashland, OR).

### Wound healing assay

Wounds were generated in confluent cell monolayers grown in 6-well plates with media containing either 0% or 5% FBS using a sterile pipette tip. Healing was observed at 0, 24, and 48 h along the scrape line and a representative field for each cell line was photographed.

### Focus formation assay

NIH3T3 cells were plated at 5 × 10^5 ^cells/well in a 6-well plate. Cells were transfected with 1 μg of pWPXLd or pWPXLd-mTrop2 using Lipofectamine 2000 (Invitrogen, Carlsbad, CA). NIH-3T3 cells expressing mTrop2 or GFP were then seeded in triplicate at 1 × 10^5 ^cells/well in a 6-well plate. Cells were allowed to grow and fed three times a week until foci with a diameter larger than 1 mm appeared. Cells were then washed twice and foci counted.

### Soft agar assay

A total of 10^4 ^Panc02-GFP, Panc02-mTrop2 cells were plated in triplicate in 6-well plates with 2 ml of growth medium containing 0.35% agar and used to overlay 4 ml layers of growth medium containing 0.7% agar. Colonies with a diameter greater than 0.2 mm were counted using a dissecting microscope.

### Mouse models

Subconfluent and stable Panc02-GFP and Panc02-mTrop2 cells were harvested and resuspended in DMEM. For the orthotopic murine model, Panc02 cells were also used. For the subcutaneous (s.c.) tumor model, 2 × 10^5 ^cells were inoculated into the right flank of 7- to 8-week-old female nude mice (National Cancer Institute-Charles River). For the orthotopic tumor model, 5 × 10^4 ^cells were injected into the pancreas of 7- to 8-week-old female nude mice. For intrapancreatic injection, mice were anesthetized with 2.5% Avertin and an incision of 1-cm was made in the left subcostal region. The spleen was exteriorized and tumor cells in a volume of 50 μl were injected into the pancreas. For the s.c. tumor model, tumor size was measured twice weekly using digital calipers and the tumor volume was calculated with the formula: tumor volume (mm^3^) = [length (mm)] × [width (mm)]^2 ^× 0.52. For the orthotopic tumor model, mice were euthanized after 14 days. Tumors were extracted and weighed. All experiments were performed according to protocols approved by the Institutional Animal Care and Use Committee at Baylor College of Medicine.

### Statistical analysis

Quantitative results are shown as mean ± SD. Statistical analysis was done using Student's *t *tests for paired data between the control and mTrop2 groups or one-way ANOVA to determine significant difference between groups. *P *< 0.05 was considered significant.

## Competing interests

The authors declare that they have no competing interests.

## Authors' contributions

RC performed cell line generation, western blotting, proliferation assays, cell cycle analysis, wound healing assay, focus formation assay, flow cytometry, soft agar assay, AP-1 SEAP assays, immunohistochemistry, subcutaneous and orthotopic mouse models and drafted manuscript. SZ helped in the subcutaneous and orthotopic models. ML and CC participated in discussion. QY participated in discussion and manuscript preparation and provided grant support for this study. All authors read and approved the final version of this manuscript.
